# Mexican Americans agree to participate in longitudinal clinical research more than non-Hispanic whites

**DOI:** 10.1186/s12889-023-16998-6

**Published:** 2023-10-20

**Authors:** Chen Chen, Xu Shi, Lynda D Lisabeth, Madeline Kwicklis, Madelyn Malvitz, Erin Case, Lewis B Morgenstern

**Affiliations:** 1 Department of Epidemiology, School of Public Health, University of Michigan, 1415 Washington Heights, Ann Arbor, MI 48109 USA; 2 Department of Biostatistics, School of Public Health, University of Michigan, Ann Arbor, MI USA; 3 Stroke Program, University of Michigan Medical School, Ann Arbor, MI USA

**Keywords:** Mexican Americans, Ethnic differences, Research participation, Trends, Stroke

## Abstract

**Background:**

The National Institutes of Health has advocated for improved minority participation in clinical research, including clinical trials and observational epidemiologic studies since 1993. An understanding of Mexican Americans (MAs) participation in clinical research is important for tailoring recruitment strategies and enrollment techniques for MAs. However, contemporary data on MA participation in observational clinical stroke studies are rare. We examined differences between Mexican Americans (MAs) and non-Hispanic whites (NHWs) participation in a population-based stroke study.

**Methods:**

We included 3,594 first ever stroke patients (57.7% MAs, 48.7% women, median [IQR] age 68 [58–79]) from the Brain Attack Surveillance in Corpus Christi Project, 2009–2020 in Texas, USA, who were approached and invited to participate in a structured baseline interview. We defined participation as completing a baseline interview by patient or proxy. We used log-binomial models adjusting for prespecified potential confounders to estimate prevalence ratios (PR) of participation comparing MAs with NHWs. We tested interactions of ethnicity with age or sex to examine potential effect modification in the ethnic differences in participation. We also included an interaction between year and ethnicity to examine ethnic-specific temporal trends in participation.

**Results:**

Baseline participation was 77.0% in MAs and 64.2% in NHWs (Prevalence Ratio [PR] 1.20; 95% CI, 1.14–1.25). The ethnic difference remained after multivariable adjustment (1.17; 1.12–1.23), with no evidence of significant effect modification by age or sex (*P*_*interaction by age*_ = 0.68, *P*_*interaction by sex*_ = 0.83). Participation increased over time for both ethnic groups (*P*_*trend*_ < 0.0001), but the differences in participation between MAs and NHWs remained significantly different throughout the 11-year time period.

**Conclusion:**

MAs were persistently more likely to participate in a population-based stroke study in a predominantly MA community despite limited outreach efforts towards MAs during study enrollment. This finding holds hope for future research studies to be inclusive of the MA population.

## Introduction

The National Institutes of Health has advocated for improved minority participation in clinical research, including clinical trials and observational epidemiologic studies since 1993 [1–3]. A high prevalence of non-participation may be the greatest threat to generalizability, and representative samples of underserved populations are a prerequisite for health equity research [4–7].

Numerous studies have reported ethnic differences in participation in clinical research and corresponding strategies to improve minority participation [8–15]. However, the study designs investigated were almost exclusively clinical trials and focused primarily on differences between Black Americans and non-Hispanic whites (NHWs) [6–11]. Studies specifically designed to evaluate ethnic differences between Mexican Americans (MAs) and NHWs participation in observational epidemiologic studies are scarce [13].

MAs are the largest and fastest growing subgroup of Latinx who are the most numerous minority group in the US [13, 16]. Given the greater stroke burden in MAs compared with NHWs and the health disparities between these groups [17–19], understanding MA participation in clinical research is important for tailoring recruitment strategies and enrollment techniques for MAs which are efficient and cost-effective.

Further, although it has been widely reported that participation in observational epidemiological studies has decreased over the past several decades [20–24], none of these studies showed ethnic-specific time trends in participation. Other studies on historical trends in ethnic differences in participation all focused on clinical trials and had mixed findings on trends in the Latinx participation, with some reporting persistent or worsening underrepresentation, others presenting increased representation over time, and all lacking data clarifying whether the changes in representation resulted from changes in eligibility, access to clinical research, or willingness to participate, or a mixture of those [9, 10, 25–27].

Therefore, in this study, we aimed to specifically examine the differences between MAs and NHWs participation in a longitudinal population-based stroke study and ethnic-specific temporal trends in participation.

## Methods

### Study design of the Brain Attack Surveillance in Corpus Christi (BASIC) Project

Data for the current study came from the BASIC Project between Jan 1, 2009 and Jan 1, 2020. BASIC is an ongoing population-based stroke study in Nueces County, Texas, which had a population of 362,294 in 2020, with 63.2% Latinx (almost exclusively MAs) who are mostly second- or third-generation US citizens and reside in the urban city of Corpus Christi [19, 28–30]. This stable, mostly nonimmigrant MA population is representative of the broader MA population in the U.S. [29].

BASIC is integrated into the Corpus Christi community to ensure equal access to study participation by ethnicity. The simple but multiple strategies employed to facilitate the integration include assembling a local research team comprised of field coordinators all indigenous to the region, hiring adequate bilingual study coordinator, and providing all project materials in Spanish and English versions. Research coordinators are trained in study recruitment and implementation processes. Additionally, Spanish-speaking individuals are approached by a bilingual research coordinator [28]. Details of the BASIC Project have been described elsewhere [19, 28].

The project was approved by the Institutional Review Boards at the University of Michigan and the 2 local hospital systems in Corpus Christi. All study participants or their proxies provided written informed consent. Given the restricted nature of the data, deidentified data will only be available upon reasonable request to the principal investigators of the BASIC Project.

### Study population

All first-ever stroke cases who were either MA or NHW identified in BASIC were included in the current study. BASIC uses active and passive surveillance to capture all possible stroke cases among residents aged ≥ 45 years. Cases are validated by fellowship-trained stroke physicians blinded to race-ethnicity and age using source documentation [30]. Active surveillance involves the identification of cases through daily screening of hospital admission logs, medical wards, and intensive care units. Passive surveillance involves identifying cases by searching hospital and emergency department discharge diagnoses, using *International Classification of Diseases*, *Ninth and Tenth Revision*, codes (ICD 430–438/I60-69).

### Baseline interview participation

Patients with stroke are approached and invited to participate in a structured baseline interview as quickly as possible after stroke onset by research coordinators. Before the interview begins, informed consent is obtained from the patient or proxy and a series of orientation questions are asked to ensure that the patient is capable of providing accurate responses. For patients unable to speak or communicate and those who cannot be consented, are comatose, unresponsive, or died before being approached, proxy interviews are done with the person who best knew the patient’s daily activities and medical history. Interviews are conducted in English or Spanish depending on patient or proxy preference. Most interviews occur in person, but can also occur via phone if necessary. We defined participation as completing the baseline interview by patient or their proxy within 4.5 months after stroke onset.

### Ethnicity and other baseline covariates

Information on race-ethnicity was ascertained from medical records with previously reported 96% agreement with self-reported ethnicity in this community [31]. Factors that were available in our database and associated with research participation based on prior literature were selected as potential confounders for our study [8, 24, 32, 33]. These prespecified potential confounders were abstracted from medical records at stroke onset and included age, sex, health insurance status, initial stroke severity (measured by National Institutes of Health Stroke Scale, NIHSS), disease histories of hypertension, high cholesterol, diabetes, atrial fibrillation, coronary artery disease, and smoking status and alcohol consumption. Additionally, a comorbidity index was included as a potential confounder, which was calculated as a sum of the following conditions consistently collected from medical records during the study period: Alzheimer’s disease or dementia, coronary artery disease or myocardial infarction, atrial fibrillation, heart failure, cancer, chronic obstructive pulmonary disease, diabetes, end-stage renal disease, epilepsy, high cholesterol, hypertension, Parkinson’s disease. Since individual-level socioeconomic status (SES) at the time of the baseline approach for participation was not available, we determined neighborhood-level SES calculated according to the patient’s census tract of residence at the time of stroke onset. Specifically, a patient’s geocoded home address at stroke onset was abstracted from the medical record and then linked to the 2010 US census tracts. We used three census tract-level variables, including measures of the proportion of disadvantage, affluence, and ethnic immigrant concentration. Specifically, neighborhood socioeconomic disadvantage was aggregated across four census indicators (proportion of female headed families with children, proportion of household with public assistance income or food stamps, proportion of families with income below the federal poverty level, and proportion of population age ≥ 16 unemployed), neighborhood affluence was summarized across three census indicators (proportion of households with income greater than $75 k, proportion of population age ≥ 16 employed in professional or managerial occupations, and proportion of adults with Bachelor’s Degree or higher), and neighborhood ethnic immigrant concentration was calculated by averaging two census indicators (proportion Hispanic and proportion foreign born) [34].

### Statistical analysis

We investigated baseline characteristics overall and by ethnicity. We calculated ethnic-specific prevalence of baseline participation in all included participants, among those who survived past baseline, and among those who had died before the baseline approach and were interviewed by a proxy. Because the date of baseline is dynamic for each patient, the subsample of those who survived past baseline was determined if patients or their proxies 1) participated in the baseline interview before patients’ death or 2) did not participate in the baseline interview but the patient survived 30 days after stroke. Among this subsample of patients, we further estimated prevalence of baseline participation by interview type (i.e. patient interviews vs proxy interviews). Similarly, the subsample of those who died before baseline approach consisted of patients who 1) had their proxies complete their baseline interview after their death or 2) died within 30 days after stroke and did not participate in baseline interviews.

To control for the possible influence of baseline survival status on ethnic differences in baseline interview participation, we examined whether age-adjusted 30-day mortality after stroke differed by ethnicity and whether prevalence of participation differed by mortality status. To examine the prevalence ratio (PR) of baseline participation comparing MAs with NHWs, we fit a series of log-binomial models including the binary ethnicity indicator as the main predictor and three different sets of prespecified potential confounders. Model 1 was adjusted for calendar year and demographics, including age, sex, and health insurance status. Model 2 was additionally adjusted for stroke severity and comorbidities and health behavior factors, which included disease histories of hypertension, high cholesterol, diabetes, atrial fibrillation, coronary artery disease, and comorbidity score, smoking status, and drinking status; and model 3 (final model) was further adjusted for neighborhood-level SES, including the measures of disadvantage, affluence, and ethnic immigrant concentration. Functional forms of continuous variables (calendar year, age, and initial stroke severity) were determined by comparing models including higher order polynomial terms (i.e. quadratic term) and models including the linear term using likelihood ratio tests. All the three continuous variables required quadratic forms. We also included an interaction term between ethnicity and age and ethnicity and sex separately in the final model to understand the potential effect modification by age or sex on ethnic differences in participation. Finally, to estimate whether ethnic differences in baseline participation changed over time, we included the interaction between calendar year and ethnicity in the final model. Because models with the interaction terms did not significantly improve the model fit, we removed them from the final model for ease of interpretation of results.

All statistical analyses were performed using SAS 9.4 (SAS Institute, Cary, NC) from October 2022 to January 2023 and all statistical tests were two-sided with type I error rate set at 0.05 being considered as statistically significant.

## Results

In total, 3,664 first-ever stroke patients were identified between Jan 1, 2009 and Jan 1, 2020. Among them, 70 (1.9%) patients had missing covariate data and were excluded, leaving 3,594 patients with 1,520 (42.3%) NHWs and 2,074 (57.7%) MAs in the analytical sample. As shown in Table 1, MAs were younger, were less likely to be insured and live in a higher SES neighborhood at baseline, whereas NHWs were less likely to have hypertension, diabetes, and more likely to have atrial fibrillation and be current smokers.

**Table 1:** Characteristics of the study population overall and by ethnicity at baseline

Characteristics^a^	All (*N* = 3,594)	NHWs (*N* = 1,520)	MAs (*N* = 2,074)
Survived	3,183 (88.6)	1,315 (86.5)	1,868 (90.1)
Demographics			
Age	68.0 (58.0–79.0)	71.5 (61.0–82.0)	66.0 (57.0–77.0)
Women	1749 (48.7)	760 (50.0)	989 (47.7)
Insured	3074 (85.5)	1365 (89.8)	1709 (82.4)
Stroke severity	4.0 (1.0–10.0)	7.3 (1.0–10.0)	7.3 (1.0–9.0)
Comorbidities			
Number of comorbidities	3.0 (1.0–4.0)	2.6 (1.0–4.0)	2.6 (2.0–4.0)
Hypertension	2864 (79.7)	1152 (75.8)	1712 (82.6)
High cholesterol	1552 (43.2)	650 (42.8)	902 (43.5)
Diabetes mellitus	1562 (43.5)	464 (30.5)	1098 (52.9)
Atrial fibrillation	497 (13.8)	292 (19.2)	205 (9.9)
Coronary artery disease	880 (24.5)	380 (10.6)	500 (13.9)
Health behaviors			
Smoking status			
Current	795 (22.1)	351 (23.1)	444 (21.4)
Former	602 (16.8)	299 (19.7)	303 (14.6)
Never	2197 (61.1)	870 (57.2)	1327 (64.0)
Excessive drinking	274 (7.6)	112 (7.4)	162 (7.8)
Neighborhood SES			
Affluence	24.4 (14.3–35.6)	32.8 (24.0–44.9)	18.1 (11.0–26.4)
Disadvantage	11.8 (7.9–15.8)	8.8 (5.8–12.7)	14.0 (10.2–17.1)
Ethnic Immigration	37.1 (28.2–47.5)	29.8 (23.0–36.9)	44.8 (35.6–49.0)

*NHWs* non-Hispanic whites, *MAs* Mexican Americans

^a^ Stroke severity was measured by National Institue of Health Stroke Scale (NIHSS). For continuous variables (age, NIHSS, neighborhood SES, number of comorbidities), because they were not normally distributed, median (interquartile range) was used. For the remaining categorical variables, numbers (percentages) were used unless stated otherwise

Table 2 displays the ethnic-specific baseline participation prevalence overall and by baseline survival status. The overall baseline participation rate was 77.0% in MAs and 64.2% in NHWs (PR 1.20; 95% CI, 1.14–1.25). Regardless of baseline survival status and interview type, participation prevalence was higher in MAs than NHWs. Median time to baseline participation was 7 days (IQR: 3–17) for those who survived and 45 days (IQR: 33–67) for those who died, but the time did not differ by ethnicity.

**Table 2:** Baseline participation for survivors vs deceased by ethnicity

Participation	All (*N* = 3,594)	Survived (*N* = 3,183)			Deceased (*N* = 411)
		Overall	Non-proxy	Proxy	
**Overall**					
Prevalence	2,572 (71.6)	2,335 (73.4)	1,784 (56.0)	551 (17.3)	237 (57.7)
Median time to baseline interview (days)	9.0 (3.0–22.0)	7.0 (3.0–17.0)	6.0 (3.0–16.0)	10.0 (4.0–21.0)	45.0 (33.0–67.0)
**NHWs**	**(*N* = 1,520)**	**(*N* = 1,315)**			**(*N* = 205)**
Prevalence	976 (64.2)	869 (66.1)	677 (51.5)	192 (14.5)	107 (52.2)
Median time to baseline interview (days)	9.0 (4.0–23.0)	7.0 (3.0–17.0)	6.0 (3.0–16.0)	11.0 (5.0–21.0)	45.0 (30.0–59.0)
**MAs**	**(*N* = 2,074)**	**(*N* = 1,868)**			**(*N* = 206)**
Prevalence	1,596 (77.0)	1,466 (78.5)	1,107 (59.3)	359 (19.2)	130 (63.1)
Median time to baseline interview (days)	8.0 (3.0–22.0)	7.0 (3.0–17.5)	6.0 (3.0–16.0)	9.0 (4.0–21.0)	44.5 (35.0–75.5)

Table 3 reports the adjusted prevalence ratios of baseline participation comparing MAs with NHWs. MAs had an 18% higher prevalence of participating in baseline interviews (PR 1.18; 95% CI, 1.13–1.23;* P* < 0.001) after adjusting for year and demographic factors including age, sex, and insurance status. The results remained similar after further adjusting for stroke severity, comorbidities, health behaviors, and neighborhood SES. In the fully adjusted model, MAs had a 17% higher prevalence of participating in baseline interviews (PR 1.17, 1.12–1.23; *P* < 0.001). There was no evidence of age or sex modifying the ethnic difference in participation (*P*_*interaction by age*_ = 0.68, *P*_*interaction by sex*_ = 0.83). Other factors associated with participation were age, insurance status, history of diabetes, and smoking status. An interquartile range increase in age was associated with a 11% lower prevalence of baseline participation (PR 0.89, 0.84–0.94). Both having insurance and having diabetes were associated with 6% lower prevalence of baseline participation. Compared with never-smokers, former smokers were 5% more likely to participate in the baseline interview (PR 1.05, 1.00–1.11) whereas no difference in participation (PR 0.96, 0.91–1.01) were found between current smokers and never-smokers.

**Table 3:** Adjusted association between ethnicity and baseline participation in the BASIC Project (2009–2020)

	**Prevalence ratio (95% CI)**		
	**Model 1**	**Model 2**	**Model 3**^**d**^
**Demographics**			
**Ethnicity (MAs vs NHWs)**	1.18 (1.13, 1.23)	1.18 (1.13, 1.24)	**1.17 (1.12, 1.23)**
**Age**^**a**^	0.91 (0.86, 0.96)	0.89 (0.84, 0.94)	**0.89 (0.84, 0.94)**
**Sex (Female vs Male)**	0.99 (0.96, 1.03)	1.00 (0.96, 1.04)	1.00 (0.96, 1.04)
**Insurance (Yes vs No)**	0.97 (0.91, 1.02)	0.94 (0.89, 1.00)	**0.94 (0.89, 1.00)**
**Stroke severity**^**b**^		1.11 (1.05, 1.18)	**1.11 (1.05, 1.18)**
**Comorbidities**			
**Number of comorbidities**		1.02 (0.99, 1.05)	1.02 (0.99, 1.04)
**Hypertension**		1.02 (0.96, 1.08)	1.02 (0.96, 1.08)
**High cholesterol**		1.03 (0.98, 1.08)	1.03 (0.98, 1.09)
**Diabetes**		0.94 (0.89, 0.99)	**0.94 (0.89, 0.99)**
**Atrial Fibrillation**		0.98 (0.91, 1.05)	0.98 (0.91, 1.06)
**Coronary artery disease**		1.00 (0.94, 1.06)	1.00 (0.94, 1.06)
**Health behaviors**			
**Smoking status**			
**Never**		Ref	Ref
**Former**		1.05 (1.00, 1.11)	**1.05 (1.00, 1.11)**
**Current**		0.96 (0.91, 1.01)	0.96 (0.91, 1.01)
**Excessive drinking (Yes vs No)**		0.97 (0.89, 1.05)	0.96 (0.89, 1.05)
**Neighborhood SES**^**c**^			
**Affluence**			1.00 (0.95, 1.06)
**Disadvantage**			0.99 (0.96, 1.03)
**Ethnic Immigration**			1.02 (0.97, 1.08)

^a^ Age was modeled in quadratic functional form and the effect of age was demonstrated as the prevalence ratio of an interquartile range increase in age among the population who has median age of the study population

^b^ Stroke severity was modeled in quadratic functional form of the natural logarithm of NIHSS plus 1 and the effect of stroke severity was demonstrated as the prevalence ratio of an interquartile range increase in NIHSS among the population who has median stroke severity of the study population

^c^ The three measures of neighborhood SES were modeled continuously and the effects were shown as the prevalence ratio of an interquartile range increase in the measure

^d^ Boldface indicates statistical significance (*P* < 0.05)

**Fig. 1:**
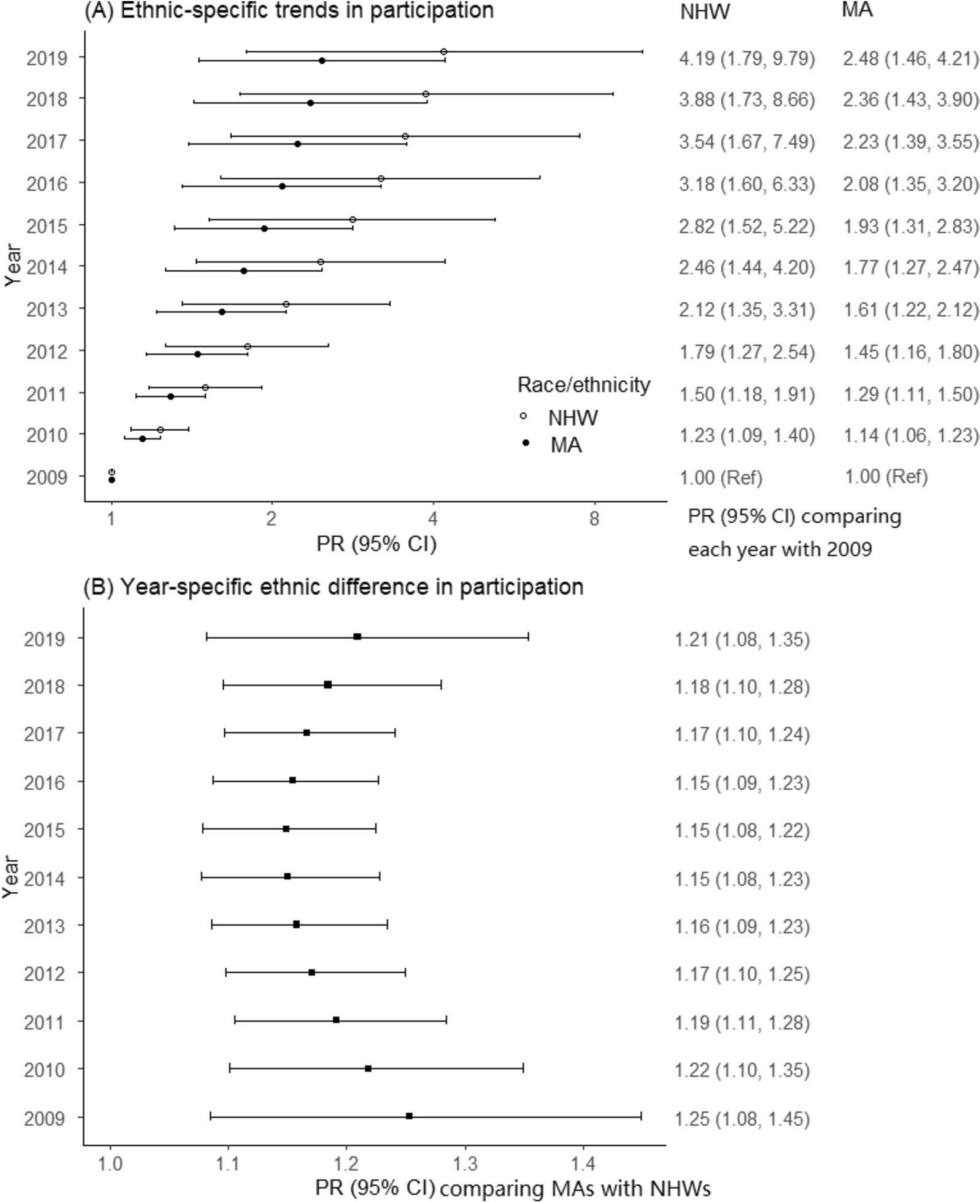
Ethnic differences in participation over time. **A** shows the ethnic-specific participation prevalence ratio comparing each year to 2009 (*P*_*interaction*_ for year*ethnicity = 0.58; *P*_*trend for MA*_ = 0.001;* P*_*trend for NHW*_ = 0.001). **B** shows the participation prevalence ratio comparing MAs to NHWs in each year. Prevalence ratios were adjusted for age, sex, health insurance status, stroke severity, disease histories of hypertension, high cholesterol, diabetes, atrial fibrillation, coronary artery disease, comorbidity score, smoking status, excessive drinking status, and neighborhood SES indicators. Functional forms of calendar year, age, and stroke severity required quadratic terms

Figure 1 shows ethnic-specific PR of baseline participation at each year compared with 2009 and year-specific PR of baseline participation comparing MAs with NHWs. For both ethnic groups, patients were significantly more likely to participate in baseline interviews over time (*P*_*trend for MA*_ = 0.001;* P*_*trend for NHW*_ = 0.001), but the disparity between MAs and NHWs did not change over time (*P*_*interaction*_ = 0.58). Compared with 2009, in 2010, NHWs were 1.23 (1.09–1.40) and MAs were 1.14 (1.06–1.23) times more likely to participate in the baseline interview respectively. Compared with NHWs, MAs were consistently more likely to participate in the baseline interview although the changes in the association between ethnicity and participation over time were U-shaped. The ethnic difference in baseline participation was the smallest (PR 1.15, 1.08–1.23) in 2015 and the largest (PR 1.25, 1.08–1.45) in 2009.

## Discussion

In this prospective biethnic population-based study with long-standing recruitment and a large sample size of stroke patients, we found that MAs were more likely to participate in the research study than NHWs. This may reflect the unique characteristics of a predominantly MA community fostering minority research participation. Our research also showed that participation increased among both ethnic groups from 2009 to 2019, which was rarely noted in previous studies, especially among the MA population. Additionally, the unique population-based design of BASIC allows for adjustment of known factors that differ by ethnicity and may influence participation in clinical research (sociodemographics, comorbidities, and health behavior factors). In our study, the ethnic difference in participation remained even after adjustment, suggesting that the noted difference was not explained by ethnic differences in potential confounders.

Findings about ethnic differences between Latinx and NHW participation in clinical research are inconsistent in the literature. In the current study, MA stroke patients had a higher baseline participation prevalence than NHWs despite the limited outreach efforts the BASIC project has made to encourage minority participation. This is consistent with the findings of another population-based study among 996 MA and NHW participants aged ≥ 65 and who agreed to cognitive screening in the same Corpus Christi community [13]. One potential reason for the excellent MA participation is the study design and aims of BASIC, which include a strong focus on MAs in the research and ensure equal access to participation through simple methods that have been suggested to affect Latinx participation in research. These methods include recruiting local MA residents as field research coordinators and hiring adequate bilingual study coordinator [14, 28].

Our current finding is also consistent with one systematic review in 2005 by Wendler et al. [12]. In this review, which included three US national representative health surveys and 13 clinical trials, Wendler et al. concluded that Latinx were at least as willing as NHWs to participate in clinical research when eligible and invited to participate [12]. Although some studies showed lower participation of Latinx compared with NHWs [9–11], it is unclear whether the ethnic disparities in participation are a result of lower eligibility rates or less willingness to participate among those Latinx. Additionally, specific diseases of interests in these previous studies were cancer or dementia and prior research has shown that minority participation may vary by specific diseases of interests [32]. Therefore, these prior findings are not directly comparable to our study, which focuses on the participation in stroke research among a predominantly MA population who were eligible and consented for our study.

The increasing overall trend in participation we observed in this population-based observational study aligns with several recent systematic reviews on diabetes, heart failure, and cancer trials reporting increasing Latinx participation over the past decades or so [9, 25, 27]. However, our results contradict the widely accepted notion that participation rates declined in observational epidemiologic studies [20–24]. The reasons for the observed increase in the current study and the discrepancy with previous observational studies are not well understood, but data from prior research was available through only 2012, and most participants in those studies were NHWs. Further, the two major challenges contributing to decreasing participation as summarized by a systematic review [32], increased refusal and difficulty finding eligible study participants, are probably not impactful factors for nonparticipation in the current study. Changes over time in methods for recruitment and sociocultural factors that may influence participation, especially minority participation, are also possible explanations [20, 32].

In this study, we also found that patients who were older, had insurance, and had diabetes were less likely to participate, while those who had more severe stroke and those who were former smokers compared with never smokers were more likely to participate. In prior research, the association between age and participation has been inconsistent with some studies reporting older persons more likely to participate [24, 35, 36] and others reporting higher participation rates among younger persons [8, 37], similar to our results. Although there were few studies that investigated the association between diabetes history and research participation, our finding of the negative association between diabetes and participation agrees with the evidence that study nonparticipants had higher disease rates and poorer health status compared with participants [36, 38]. However, the reason why patients with more severe stroke were more likely to participate needs to be further investigated. One possible explanation is that patients with more severe stroke may be more likely to rely on proxies to complete the baseline interview and our results suggested that prevalence of baseline participation was higher among proxies than patient themselves.

Despite the evidence that sociodemographics, comorbidities, and health behavior factors are associated with participation and the ethnic differences in these factors in the BASIC Project [8, 24, 32, 33], the ethnic difference in participation in our study remained after adjusting for these factors. It is possible that the ethnic difference in participation we observed was mainly driven by the differential participation in certain demographic subgroups, however, we found that the ethnic difference in participation was similar across age and sex groups. Choice of study site may also have an important impact on who is more likely to enroll. MAs in Corpus Christi, who are predominantly second and third generation US born citizens, have resided in the community longer, on average, than NHWs [39]. Further, MAs are the predominant race-ethnicity group in this Corpus Christi community with 63.2% Latinx (almost exclusively MAs).

Our results may provide insights into the design and implementation of future observational stroke studies, particularly in MA populations, such as engaging with individuals from the communities in a culturally sensitive way and ensuring equal access to participation by equally informing and inviting minority groups. However, our study is not without limitations. Even though we adjusted for major risk factors for nonparticipation in our statistical analysis, our results are still susceptible to residual confounding. For example, we were unable to adjust for language spoken due to a lack of data. Nevertheless, we can assume that very few patients in our cohort cannot speak English as BASIC is based on a mostly nonimmigrant community, and therefore the influence of language spoken on ethnic differences in participation should be negligible. Another limitation is that we may not be able to generalize our findings to immigrant MAs or other race-ethnic groups since BASIC is a single community of predominantly nonimmigrant MAs. Future studies assessing participation in observational epidemiologic research in diverse populations are needed.

## Conclusion

In this population-based stroke study, MAs were persistently more likely to participate than NHWs despite limited outreach efforts towards MAs during study enrollment. This finding holds hope for future research studies to be inclusive of the MA population.

## Data Availability

Given the restricted nature of the data, deidentified data will only be available upon reasonable request to the principal investigators of the BASIC Project (lmorgens@umich.edu).

## Abbreviations

MAs: Mexican Americans
NHWs: Non-Hispanic whites
BASIC: Brain Attack Surveillance in Corpus Christi
SES: Socioeconomic Status
PR: Prevalence Ratio
